# Metabolic Syndrome and Psoriasis: Mechanisms and Future Directions

**DOI:** 10.3389/fimmu.2021.711060

**Published:** 2021-07-23

**Authors:** Yan Hao, Ya-juan Zhu, Song Zou, Pei Zhou, Ya-wen Hu, Qi-xiang Zhao, Lin-na Gu, Hao-zhou Zhang, Zhen Wang, Jiong Li

**Affiliations:** ^1^ State Key Laboratory of Biotherapy and Cancer Center, West China Hospital, West China Medical School, Sichuan University, and Collaborative Innovation Center for Biotherapy, Chengdu, China; ^2^ Department of Biotherapy and Cancer Center, State Key Laboratory of Biotherapy, West China Hospital, Sichuan University, Chengdu, China; ^3^ Department of Cardiology, West China Hospital, Sichuan University, Chengdu, China; ^4^ Department of Liver Surgery & Liver Transplantation, State Key Laboratory of Biotherapy and Cancer Center, West China Hospital, Sichuan University and Collaborative Innovation Center of Biotherapy, Chengdu, China; ^5^ Laboratory of Liver Surgery, West China Hospital, Sichuan University, Chengdu, China

**Keywords:** psoriasis, metabolic syndrome, gut microbiota, insulin resistance, autoimmunity, obesity

## Abstract

Psoriasis is an immune-mediated systemic disease with associated comorbidities, including metabolic syndrome (MetS) which contributes substantially to premature mortality in patients with psoriasis. However, the pathological mechanisms underlying this comorbidity are unclear. Studies have shown that the pathological parameters of psoriasis mediate the development of MetS. We reviewed the potential mechanisms which mediate the association between psoriasis and MetS, including endoplasmic reticulum stress, pro-inflammatory cytokine releases, excess production of reactive oxygen species, alterations in adipocytokine levels and gut microbiota dysbiosis. Here, we highlight important research questions regarding this association and offer insights into MetS research and treatment.

## Introduction

**Figure 1:**
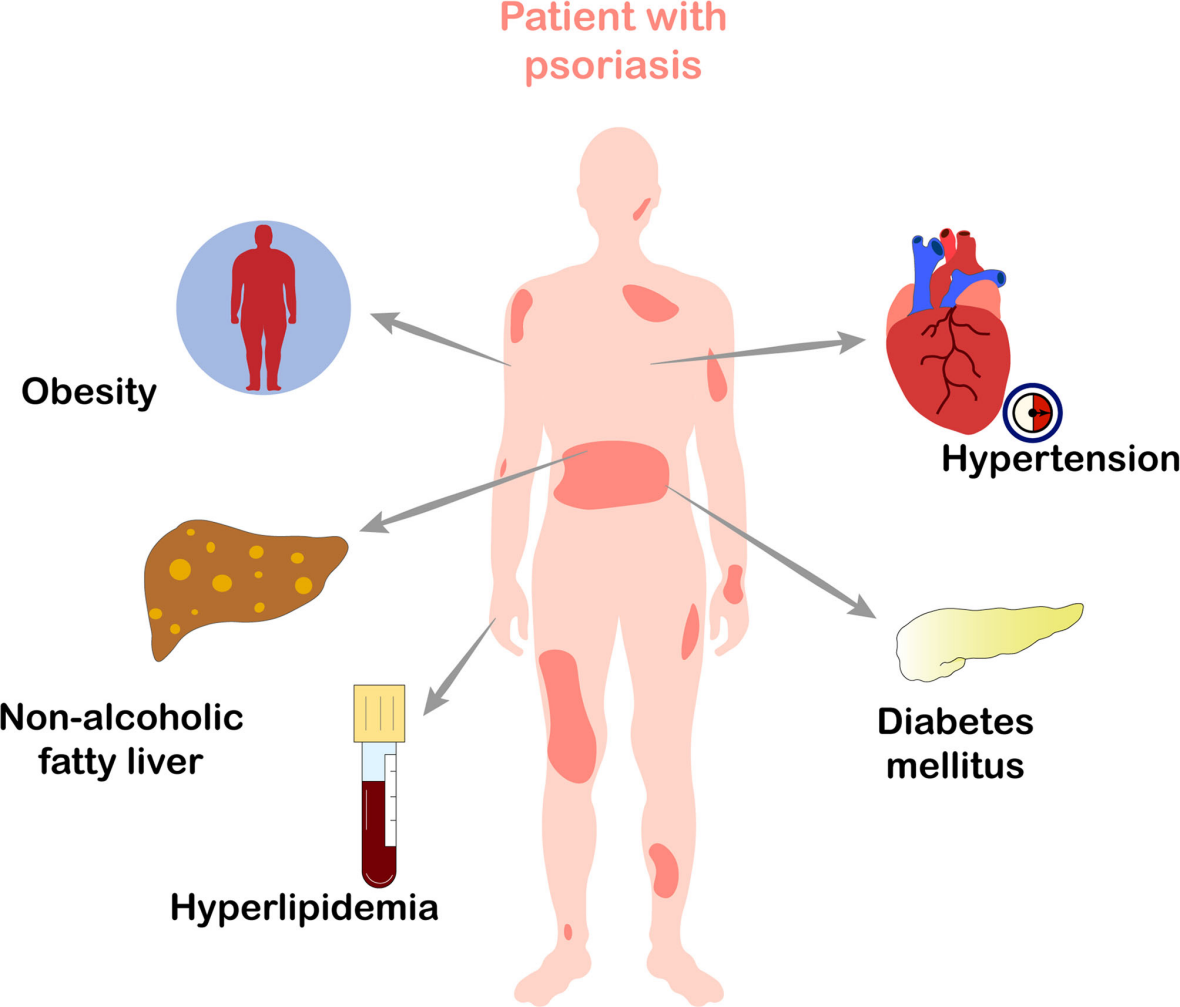
Metabolic diseases which frequently occur in patients with psoriasis. Psoriasis is often associated with obesity, hypertension, diabetes mellitus, hyperlipidemia, and obesity-associated non-alcoholic fatty liver disease, all of which belong to metabolic syndrome.

Psoriasis, one of the most common chronic, recurrent, and inflammatory skin diseases, affects 2~3% of the total world population (1). There are several clinical cutaneous manifestations of psoriasis. The disease most commonly presents as chronic, symmetrical, erythematous, scaling papules and plaques (2). Pathologically, epidermal hyperproliferation and parakeratosis are the main histological features of psoriasis. Notably, increased release of pro-inflammatory cytokines and the chronic activation of innate and adaptive immune systems result in long-term damage to multiple tissues and organs of patients with psoriasis (3). Psoriasis is a systemic disease that is associated with multiple comorbidities, such as psoriatic arthritis, Crohn’s disease, cancer, depression, cardiovascular disease (CVD) (4), and metabolic syndrome (MetS). Among these, MetS is one of the most common and important comorbidities (5–8). An increasing number of clinical studies have confirmed that psoriasis is often related with MetS, such as obesity, hypertension, diabetes mellitus, hyperlipidemia, and obesity-associated non-alcoholic fatty liver disease (NAFLD) (**Figure 1**) (9–15). Another study views that compared to patients with milder psoriasis, those with more severe psoriasis have greater hazard of MetS (16). MetS directly increases the risk of CVD and premature mortality in patients with psoriasis (17), thus substantially reducing their life expectancy. Therefore, it is critical to understand the exact mechanisms underlying the relationship between psoriasis and MetS.

Recently, the prevalence of MetS in patients with psoriasis has attracted the attention of researchers. The precise mechanisms underlying the association between psoriasis and MetS remain unknown. Therefore, in this review, we have discussed these mechanisms, particularly, the pathogenic factors possibly involved, including endoplasmic reticulum (ER) stress in multiple cells, pro-inflammatory cytokine profiles, excess production of reactive oxygen species (ROS), alterations in adipocytokine levels, and gut microbiota dysbiosis. This review highlights the previously established and the emerging important mechanisms that link psoriasis with MetS.

## Pro-Inflammatory Cytokines Linking Psoriasis and MetS

The hallmark of psoriasis is sustained inflammation (18). The pathogenesis of psoriasis has involvement of dynamic interactions between multiple cell types and cytokines (19). Th17 cells produce several cytokines, such as IL-17 (IL-17A/IL-17F), tumor necrosis factor-α (TNF-α), and IL-22 (4), which induce altered differentiation and hyperproliferation of keratinocytes. Therefore, Th17 cells play a predominant role in the pathogenesis of psoriasis and are indicators of an increased risk of psoriasis. Moreover, pro-inflammatory cytokines are implicated in many diseases, including obesity, diabetes mellitus, hypertension, NAFLD, and hyperlipidemia (20–23). It is well-known that tissue inflammation plays a critical role in insulin resistance (IR) (24–26). IR is the key primary defect underlying the development of type 2 diabetes (T2D), and it is also a central component of MetS (27).

IL-17 plays a crucial role in inflammation, IR, and T2D, indicating that it is a potential mediator linking MetS and psoriasis (28). It promotes a vascular inflammatory response and plays a critical role in angiotensin II-induced hypertension and vascular dysfunction (29). Previous studies reported that serum IL-17 levels were significantly elevated in subjects with MetS and Type 1 diabetes compared to health group (30, 31). Secukinumab, anti-IL-17A monoclonal antibody, is an effective biological agent for the treatment of plaque psoriasis. In patients with higher response for secukinumab, mean body weight, waist circumference, and BMI consistently decreased (32). Furthermore, combined administration of anti-IL-17A monoclonal antibody (secukinumab and ixekizumab) reduced fasting glucose levels in imiquimod treated mice and improved hyperglycemia in patients with psoriasis (33), suggesting that IL-17 may be a key cytokine linking psoriasis and hyperglycemia (34).

TNF is also closely associated with the pathogenesis of psoriasis. The Food and Drug Administration has approved efficacious TNF inhibitors for the treatment of moderate and severe plaque psoriasis, including infliximab, adalimumab, and etanercept (35). Patients with psoriasis who have administration of anti-TNF drugs often show an improvement in MetS. For patients with psoriasis, it has been demonstrated that the treatment with etanercept or adalimumab improved metabolic parameters including blood lipid and glucose levels and systolic and diastolic blood pressure (36). Another study further confirmed that anti-TNF treatment improves the metabolic profile of patients with psoriasis by downregulating their total cholesterol and low-density lipoprotein (LDL) levels (37). Mechanistically, TNF-α inhibits THP-1 cell uptake of oxidized LDL, thus increasing the extracellular accumulation of oxidized LDL (38). Additionally, the treatment with adalimumab significantly improves insulin sensitivity in patients with moderate-to-severe plaque psoriasis (39). TNF-α directly contributes to IR (40, 41) by activating stress kinases, such as IκB kinase, c-Jun N-terminal kinase, and p38 mitogen-activated protein kinase in muscle and fat cells, thereby blocking insulin signal transduction (42, 43). In some views, anti-TNF-α antibody is supposed as the first-line treatment for psoriasis with metabolic syndrome (44). In contrast, a view points out that the treatment with anti-TNF (infliximab and adalimumab) leads to no significant changes in insulin sensitivity or fasting glucose levels, but increased body fat (45). This may be due to the limited studied population, and only men were included.

**Figure 2:**
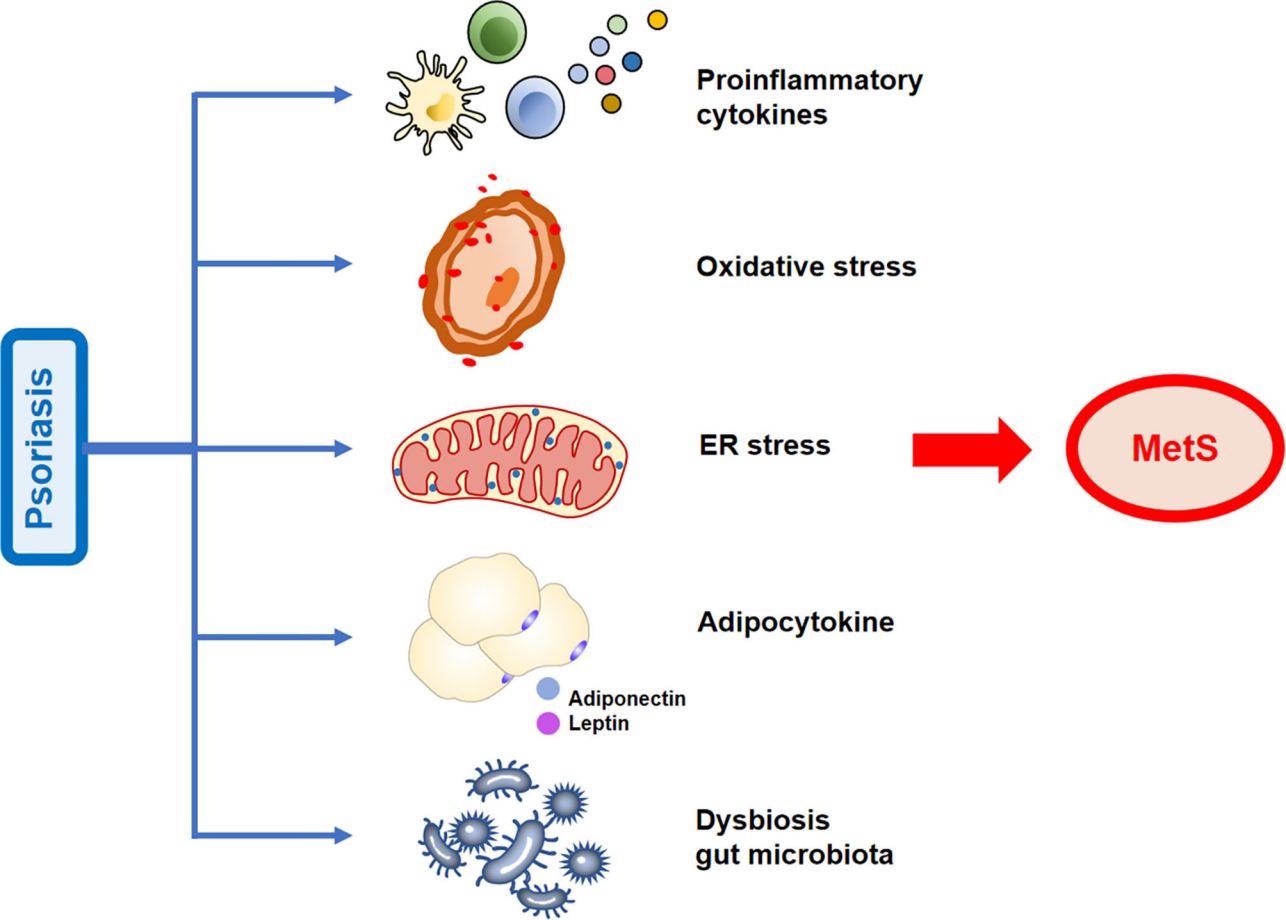
Possible mechanisms linking psoriasis and metabolic syndrome (MetS). The increased release of pro-inflammatory cytokines, adipose tissue secretory adipocytokines, activation of oxidative stress states, increased endoplasmic reticulum (ER) stress, and gut microbiota dysbiosis observed in psoriasis contribute to the development of MetS.

Moreover, serum IL-1β, IL-6, and IL-22 levels were significantly upregulated in patients with T2D (46) and obese individuals (47, 48). Notably, deletion of IFN-γ improves IR and metabolic parameters in diet-induced obesity models (49). The above-mentioned studies have demonstrated that the immune system is closely linked to metabolic disorders. Pro-inflammatory cytokines may link psoriasis with MetS (**Figure 2**).

## Adipocytokines Linking Psoriasis and MetS

Adipose tissues secrete adipocytokines that modulate organ functions and lipid metabolism. Leptin and adiponectin, two classical adipokines, are well-established endocrine hormones that act on specific receptors of remote target organs (50). Adiponectin is an insulin sensitizer that ameliorates IR and regulates glucose and lipid metabolism by binding to its receptors, AdipoR1 and AdipoR2 (51). This might be due to reduction in ectopic lipids in the liver and muscle (52). Adiponectin induces an increase in serum high-density lipoprotein (HDL) and down-regulates serum triglycerides through enhanced catabolism of triglyceride-rich lipoproteins (53). Adiponectin ameliorates obesity-induced NAFLD by interacting with hepatic peroxisome proliferator-activated receptors (54).

Several studies have demonstrated that low adiponectin concentrations in patients with psoriasis may contribute to the development of MetS. Of note, the serum adiponectin level is negatively associated with the TNF-α and IL-6 levels (55). TNF-α can impair adiponectin multimerization, consequently decreasing adiponectin secretion (56, 57). This might be a reasonable explanation for the lower adiponectin concentrations in patients with psoriasis compared to those of controls. Moreover, multiple studies postulate that adiponectin links the pathological processes of psoriasis and obesity (58). A meta-analysis has shown that patients with psoriasis exhibit low levels of adiponectin (59). Compared to patients with psoriasis without metabolic abnormalities, patients with psoriasis and MetS or high body mass index have significantly lower adiponectin levels. The psoriasis area and severity index (PASI) score (60) are negatively correlated with adiponectin levels (61, 62). Overall, low serum adiponectin levels in patients with psoriasis may be the link to MetS.

Leptin is a critical hormonal regulator of metabolism, and leptin concentrations are directly associated with the subsequent development of metabolic disorders such as IR, T2D, and CVD (63, 64). The reduction of plasma leptin levels in obese individuals can restore hypothalamic leptin sensitivity, then effectively enhancing insulin sensitivity, reduces weight gain (65). Increased leptin levels are observed in obese people and in patients with psoriasis (66), and are positively correlated with severity of psoriasis (67). The systemic anti-inflammatory drug, acitretin, used to treat psoriasis, reduces leptin levels (68). In addition, leptin is an important signaling transducer which may link obesity and psoriasis (69). One study reported that leptin levels were higher in obese patients with psoriasis than those of normal-weight patients (70). Moreover, leptin level is affected by IL-17 (71), which might explain the higher leptin concentrations observed in patients with psoriasis than controls. These studies suggest that high leptin levels may be an important factor in psoriasis-associated metabolic diseases.

Moreover, recent studies have hypothesized that several other important adipokines, such as retinol-binding protein 4, fetuin-A, and lipocalin-2 are mediators of obesity in psoriasis (58). Adipokines could serve as a crucial link in the causal relationship between psoriasis and MetS (**Figure 2**) and may serve as biomarkers for determining the risk of developing psoriasis comorbidities.

## Oxidative Stress Status in Patients with Psoriasis and the Association with MetS

Oxidative stress is the dysregulation between the production of ROS and endogenous antioxidant defense mechanisms, which causes protein and lipid peroxidation, DNA damage, and cellular dysfunction, eventually leading to cell death (72). Increased oxidative stress in adipocytes is one of pathological mechanisms of obesity-associated metabolic diseases (73, 74). Thus, reducing ROS production can increase insulin sensitivity and alleviate hyperlipidemia, hepatic steatosis, and IR (75, 76). Additionally, a positive correlation has been established between oxidative stress and low HDL levels (77). Furthermore, LDL-related dyslipidemia and impaired fasting glucose are associated with increased oxidative stress (78). Quantitative combination of natural antioxidants (vitamins C and E) prevents MetS by reducing oxidative stress (79). These results indicate that a pro-oxidant/antioxidant imbalance plays an important role in MetS development (80).

Numerous evidences support that increased ROS production and oxidative stress status are implicated in the progression of psoriasis. Studies have revealed that in the serum/plasma and blood cells of patients with psoriasis, oxidative damage markers increased (81–87). In addition, oxidative stress markers and PASI score have a positive correlation (88). The salivary total oxidative status and oxidative stress index may serve as potential diagnostic biomarkers for plaque psoriasis (89). Of note, the activated neutrophils/monocytes that generate oxidative damage may be the main source of oxidative stress in psoriasis. Increased oxidative stress in psoriasis may be caused by an insufficient antioxidant system (90). These evidences suggest that oxidative stress may contribute to the development of MetS in patients with psoriasis (**Figure 2**).

## Endoplasmic Reticulum Stress Links Psoriasis with MetS

ER is an important organelle with a vast membranous network in eukaryotic cells (91, 92). The ER has many cellular functions, including protein synthesis, folding, and transport, lipid and steroid synthesis, carbohydrate metabolism and calcium storage (93–96). The altered functions of ER can result in the accumulation of unfolded or misfolded proteins, which is a cellular condition named ER stress (97). Prolonged ER stress is a critical factor in the pathogenesis of MetS (98, 99). Inositol-requiring enzyme 1, an ER stress sensor, induces malfunction in both brown and beige fat, eventually leading to obesity (100). Treatment with the ER stress inhibitor (tauroursodeoxycholate) can improve metabolic parameters in MetS rat and mitigate the MetS-induced cardiovascular complications (101). Reducing ER stress can alleviate IR (102–105). Specifically, the interfered transport of newly synthesized insulin proreceptors from ER to the plasma membrane can inhibit the proteolytic maturation of insulin proreceptors. Consequently, the insulin signaling was broken by consuming the insulin receptors on cell surface (106).

Studies have shown that pro-inflammatory mediators such as TNF-α, IL-1β, IL-17A, and IFN-γ contribute to the induction of ER stress in multiple immunocytes such as macrophages and T cells (107–110). ER stress can be found in patients with psoriasis. Moreover, the over-expressed ER stress-associated proteins, including binding immunoglobulin heavy-chain protein, C/EBP homologous protein, and X-box binding protein 1 in the epidermis of patients with psoriasis vulgaris suggests that ER stress is increased in the keratinocytes of these patients (111). One study reported increased expression of ER stress marker GRP78/BIP in the subcutaneous fat tissues of imiquimod-induced psoriasiform in diabetic obese mice (112). Above evidences show increased ER stress is involved in psoriasis.

Overall, due to the common emergence of ER stress in both MetS and psoriasis, we may speculate that ER stress mediates their frequent co-occurrence (**Figure 2**). However, there is less evidence on ER stress in the association between MetS and psoriasis. The ER stress in psoriasis that promotes MetS is expected to elucidate in further research.

## Gut Microbiota in Patients with Psoriasis and its Association with MetS

In the bodies of adult mammals, skin, oral mucosa and gastrointestinal tract are heavily colonized by microbiota, with the largest population found in the colon. Microorganisms have variable relationships with their hosts and exist as mutualists, symbionts, or pathobionts (113). The gut microbiome plays important roles in host immunity, metabolism, and the production of numerous compounds that influence the host (114, 115). There is an emerging interest in metabolic health and gut microbiome dysbiosis. After the transplantation of metformin-treated human microbiome into germ-free mice with glucose intolerance, the glucose defects were corrected (116). Moreover, gut microbiota specifically controlled the expression of microRNAs in white adipocytes, controlling adiposity and insulin sensitivity in mice (117). Overall, these studies suggest that the gut microbiota regulates host metabolism and obesity.

Numerous studies have demonstrated the role of *Akkermansia muciniphila* in preventing obesity-associated metabolic disorders in both humans and animal models (118–121). Importantly, the intestinal microbes of patients with psoriasis show significant differences from those of healthy subjects (122–124). Notably, a previous study found that the abundance of *A. muciniphila* was significantly decreased in patients with psoriasis (125). Overall, compared with healthy controls, patients with psoriasis have a different specific intestinal microbiome. Therefore, we hypothesize that MetS in patients with psoriasis may be related to changes in the richness of specific flora (**Figure 2**).

The intestinal microbiota plays critical roles in preserving epithelial barrier integrity, forming a mucosal immune system to battle with exogenous pathogens (126, 127). There are differences in intestinal permeability between individuals with and without T2D (128, 129). The disruption of barrier integrity is closely related with the emergence of metabolic disorder, such as obesity and T2D (127–130). Alleviated metabolic endotoxemia and enhanced intestinal barrier function causes significant weight loss and improves IR in diet-induced obese mice (131). The loss of intestinal barrier can cause the bacteria translocation and produce endotoxins or harmful metabolites, then induce systemic inflammation and aggravate MetS (132). For instance, elevated bacterial lipopolysaccharides in the circulation and organs activate the transcription of cytokines *via* toll-like receptor 4, promoting IR and metabolic diseases (133). Thus, the injured mucosal barrier induced by the gut microbiota involves in the development of MetS.

It has been found that barrier integrity injury and bacterial translocation are involved in the development of psoriasis (134). Bacterial DNA was detected in the peripheral blood of patients with psoriasis (135). In patients with moderate-to-severe psoriasis, serum markers of intestinal barrier integrity injury increased (136). For example, intestinal fatty acid binding protein, a biomarker of intestinal barrier damage, significantly elevated in patients with psoriasis compared to that in controls (137). From these studies, bacterial translocation may occur in psoriasis (138). Therefore, intestinal barrier impairment and bacterial translocation caused by dysbiosis of the gut microbiota may explain pathologically metabolic diseases in patients with psoriasis. Dysregulated gut microbiota in patients with psoriasis may be a novel therapeutic target in MetS.

## Perspective

Accumulating evidence suggests there is a relationship between psoriasis and increased risks of MetS. However, major gaps in understanding of MetS in patients with psoriasis remain. In this review, we summarized numerous studies that links psoriasis and MetS. We assume that the emergence of some factors, including ER stress, pro-inflammatory cytokine releases, excess production of ROS, alterations in adipocytokine levels and gut microbiota dysbiosis, may be predictors of MetS in patients with psoriasis.

Specifically, it seems that the pathogenic pathways in psoriasis and MetS have considerable overlap. Thus, there is a possible interaction between the psoriasis and MetS. Psoriasis and MetS both show the chronic inflammatory state (139). Notably, some inflammatory factors, such as IL-17 and TNF, can both mediate the occurrence of psoriasis and MetS. Besides, adipocytokine, a vital meditator of MetS, can regulate body metabolism meanwhile contribute to the development of a pro-inflammatory state. Subsequent studies should focus on the causal relationship between the common pathogenic factors and psoriasis with MetS. Furthermore, the role of Th17-derived cytokines in the pathogenesis of psoriasis and MetS is both increasingly recognized. Anti-IL-17 agents or TNF inhibitors improved the metabolic disorder when treat psoriasis. Thus, further long-term and large-scale studies are warranted to identify whether anti-IL-17 agents or TNF inhibitors have benefits on psoriasis with MetS. Despite the pathological mechanism of MetS remains incompletely understood, oxidative stress and ER stress are considered as leading causes and can be therapeutically targeted (140, 141). In order to underly the pathophysiological mechanisms psoriasis and MetS, more connections from the complex molecular regulatory network should be established through muti-omics analysis. Future investigations should aim to determine the elaborate upstream and downstream signaling pathways that activate ER stress and oxidative stress in psoriasis complicated with MetS. What’s more, the dysregulated gut microbiota may become a novel therapeutic target in patients with psoriasis. The oral supplementation with *A. muciniphila* should be applied to investigate the effects on metabolic abnormalities in patients and or animal models with psoriasis. In addition, other possible targeted microbiotas should be screened in psoriasis and MetS through genomics and metabolomics. These selected microbiotas could be used as a biological marker for monitoring the MetS in psoriasis.

Collectively, our review implies that administration of MetS is of importance in clinical management of patients with psoriasis in the future. Elucidating the mechanisms linking MetS and psoriasis could provide potential new therapeutic targets and specific strategies to combat MetS in psoriasis, even other autoimmune disease, such as systemic lupus erythematosus and psoriatic arthritis.

## Author Contributions

YH collected and reviewed the literature and wrote the manuscript. SZ and Y-jZ wrote and revised the manuscript. PZ rechecked the manuscript and put forward meaningful comments on it. Q-xZ and Y-wH assisted in drawing. Y-wH, L-nG and H-zZ revised the manuscript. ZW and JL designed the main study and reviewedthis manuscript. All authors have read and approved the final submitted manuscript.

## Funding

This work was supported by the National Natural Science Foundation of China (81673061 to J. Li, 81472650 to J. Li, 31271483 to J. Li, 81703132 to Z. Wang), the National Science and Technology Major Project (2018ZX09303006-001-006 to J. Li and 2019ZX09201004-003 to J. Li), the Key Research and Development Program of Sichuan Province [2020YFS0271], the Applied Basic Research Program of Sichuan Province [2021YJ0420], the Postdoctoral Fund for West China Hospital [2019HXBH075], the Fundamental Research Funds for the Central Universities [2019SCU12041, the Postdoctoral Foundation of Sichuan University].

## Conflict of Interest

The authors declare that the research was conducted in the absence of any commercial or financial relationships that could be construed as a potential conflict of interest.

## Publisher’s Note

All claims expressed in this article are solely those of the authors and do not necessarily represent those of their affiliated organizations, or those of the publisher, the editors and the reviewers. Any product that may be evaluated in this article, or claim that may be made by its manufacturer, is not guaranteed or endorsed by the publisher.

## References

[1] Scher JU Ogdie A Merola JF Ritchlin C. Preventing Psoriatic Arthritis: Focusing on Patients With Psoriasis at Increased Risk of Transition. Nat Rev Rheumatol (2019) 15:153–66. 10.1038/s41584-019-0175-0 30742092

[2] Mahil SK Capon F Barker JN. Update on Psoriasis Immunopathogenesis and Targeted Immunotherapy. Semin Immunopathol (2016) 38:11–27. 10.1007/s00281-015-0539-8 26573299 PMC4706579

[3] Deng Y Chang C Lu Q. The Inflammatory Response in Psoriasis: A Comprehensive Review. Clin Rev Allergy Immunol (2016) 50:377–89. 10.1007/s12016-016-8535-x 27025861

[4] Boehncke WH Schon MP. Psoriasis. Lancet (2015) 386:983–94. 10.1016/S0140-6736(14)61909-7 26025581

[5] Takeshita J Grewal S Langan SM Mehta NN Ogdie A Van Voorhees AS. Psoriasis and Comorbid Diseases: Epidemiology. J Am Acad Dermatol (2017) 76:377–90. 10.1016/j.jaad.2016.07.064 PMC5731650 28212759

[6] Peralta C Hamid P Batool H Al Achkar Z Maximus P. Psoriasis and Metabolic Syndrome: Comorbidities and Environmental and Therapeutic Implications. Cureus (2019) 11:e6369. 10.7759/cureus.6369 31938651 PMC6957052

[7] Voiculescu VM Lupu M Papagheorghe L Giurcaneanu C Micu E. Psoriasis and Metabolic Syndrome–Scientific Evidence and Therapeutic Implications. J Med Life (2014) 7:468–71. PMC4316120 25713604

[8] Cohen AD Gilutz H Henkin Y Zahger D Shapiro J Bonneh DY. Psoriasis and the Metabolic Syndrome. Acta Derm Venereol (2007) 87:506–9. 10.2340/00015555-0297 17989888

[9] Choudhary S Pradhan D Pandey A Khan MK Lall R Ramesh V. The Association of Metabolic Syndrome and Psoriasis: A Systematic Review and Meta-Analysis of Observational Study. Endocr Metab Immune Disord Drug Targets (2020) 20:703–17. 10.2174/1871530319666191008170409 31595859

[10] Coto-Segura P Eiris-Salvado N Gonzalez-Lara L Queiro-Silva R Martinez-Camblor P Maldonado-Seral C. Psoriasis, Psoriatic Arthritis and Type 2 Diabetes Mellitus: A Systematic Review and Meta-Analysis. Br J Dermatol (2013) 169:783–93. 10.1111/bjd.12473 23772556

[11] Armstrong AW Harskamp CT Armstrong EJ. The Association Between Psoriasis and Obesity: A Systematic Review and Meta-Analysis of Observational Studies. Nutr Diabetes (2012) 2:e54. 10.1038/nutd.2012.26 23208415 PMC3542430

[12] Rodriguez-Zuniga MJM Garcia-Perdomo HA. Systematic Review and Meta-Analysis of the Association Between Psoriasis and Metabolic Syndrome. J Am Acad Dermatol (2017) 77:657–66.e658. 10.1016/j.jaad.2017.04.1133 28917453

[13] Candia R Ruiz A Torres-Robles R Chavez-Tapia N Mendez-Sanchez N Arrese M. Risk of non-Alcoholic Fatty Liver Disease in Patients With Psoriasis: A Systematic Review and Meta-Analysis. J Eur Acad Dermatol Venereol: JEADV (2015) 29:656–62. 10.1111/jdv.12847 25418531

[14] Caroppo F Galderisi A Moretti C Ventura L Fortina BA. Prevalence of Psoriasis in a Cohort of Children and Adolescents With Type 1 Diabetes. J Eur Acad Dermatol Venereol: JEADV (2021). 10.1111/jdv.17318 33914987

[15] Di Costanzo L Fattorusso V Mozzillo E Patrì A Di Caprio R De Nitto E. Psoriasis in Children With Type 1 Diabetes: A New Comorbidity to be Considered? Acta Diabetologica (2017) 54:803–4. 10.1007/s00592-017-1000-3 28500467

[16] Armstrong AW Harskamp CT Armstrong EJ. Psoriasis and Metabolic Syndrome: A Systematic Review and Meta-Analysis of Observation Studies. J Am Acad Dermatol (2013) 68:654–62. 10.1016/j.jaad.2012.08.015 23360868

[17] Kastorini CM Panagiotakos DB Georgousopoulou EN Laskaris A Skourlis N Zana A. Metabolic Syndrome and 10-Year Cardiovascular Disease Incidence: The ATTICA Study. Nutr Metab Cardiovasc Dis (2016) 26:223–31. 10.1016/j.numecd.2015.12.010 26803591

[18] Rendon A Schakel K. Psoriasis Pathogenesis and Treatment. Int J Mol Sci (2019) 20:1–28. 10.3390/ijms20061475 PMC6471628 30909615

[19] Boehncke WH. Etiology and Pathogenesis of Psoriasis. Rheum Dis Clin North Am (2015) 41:665–75. 10.1016/j.rdc.2015.07.013 26476225

[20] Donath MY. Targeting Inflammation in the Treatment of Type 2 Diabetes: Time to Start. Nat Rev Drug Discov (2014) 13:465–76. 10.1038/nrd4275 24854413

[21] Schaffler A Buechler C. CTRP Family: Linking Immunity to Metabolism. Trends Endocrinol Metab (2012) 23:194–204. 10.1016/j.tem.2011.12.003 22261190

[22] Odegaard JI Chawla A. Pleiotropic Actions of Insulin Resistance and Inflammation in Metabolic Homeostasis. Science (2013) 339:172–7. 10.1126/science.1230721 PMC3725457 23307735

[23] Chan KL Cathomas F Russo SJ. Central and Peripheral Inflammation Link Metabolic Syndrome and Major Depressive Disorder. Physiol (Bethesda) (2019) 34:123–33. 10.1152/physiol.00047.2018 PMC6586832 30724127

[24] Mathis D. Immunological Goings-on in Visceral Adipose Tissue. Cell Metab (2013) 17:851–9. 10.1016/j.cmet.2013.05.008 PMC4264591 23747244

[25] Lee YS Wollam J Olefsky JM. An Integrated View of Immunometabolism. Cell (2018) 172:22–40. 10.1016/j.cell.2017.12.025 29328913 PMC8451723

[26] Donath MY Meier DT Boni-Schnetzler M. Inflammation in the Pathophysiology and Therapy of Cardiometabolic Disease. Endocr Rev (2019) 40:1080–91. 10.1210/er.2019-00002 PMC6624792 31127805

[27] Tilg H Moschen AR. Inflammatory Mechanisms in the Regulation of Insulin Resistance. Mol Med (2008) 14:222–31. 10.2119/2007-00119.Tilg PMC2215762 18235842

[28] Abdel-Moneim A Bakery HH Allam G. The Potential Pathogenic Role of IL-17/Th17 Cells in Both Type 1 and Type 2 Diabetes Mellitus. BioMed Pharmacother (2018) 101:287–92. 10.1016/j.biopha.2018.02.103 29499402

[29] Madhur MS Lob HE McCann LA Iwakura Y Blinder Y Guzik TJ. Interleukin 17 Promotes Angiotensin II-Induced Hypertension and Vascular Dysfunction. Hypertension (2010) 55:500–7. 10.1161/HYPERTENSIONAHA.109.145094 PMC2819301 20038749

[30] Demir E Harmankaya NO Kirac Utku I Aciksari G Uygun T Ozkan H. The Relationship Between Epicardial Adipose Tissue Thickness and Serum Interleukin-17a Level in Patients With Isolated Metabolic Syndrome. Biomolecules (2019) 9:1–12. 10.3390/biom9030097 PMC6468684 30862094

[31] Baharlou R Ahmadi-Vasmehjani A Davami MH Faraji F Atashzar MR Karimipour F. Elevated Levels of T-Helper 17-Associated Cytokines in Diabetes Type I Patients: Indicators for Following the Course of Disease. Immunol Invest (2016) 45:641–51. 10.1080/08820139.2016.1197243 27611173

[32] Pinter A Gerdes S Papavassilis C Reinhardt M. Characterization of Responder Groups to Secukinumab Treatment in Moderate to Severe Plaque Psoriasis. J Dermatolog Treat (2020) 31:769–75. 10.1080/09546634.2019.1626973 31287332

[33] Ikumi K Odanaka M Shime H Imai M Osaga S Taguchi O. Hyperglycemia Is Associated With Psoriatic Inflammation in Both Humans and Mice. J Invest Dermatol (2019) 139:1329–38.e1327. 10.1016/j.jid.2019.01.029 30776434

[34] Gardner LCS Grantham HJ Reynolds NJ. IL-17 May Be a Key Cytokine Linking Psoriasis and Hyperglycemia. J Invest Dermatol (2019) 139:1214–6. 10.1016/j.jid.2019.02.038 31126427

[35] Chima M Lebwohl M. TNF Inhibitors for Psoriasis. Semin Cutan Med Surg (2018) 37:134–42. 10.12788/j.sder.2018.039 30215629

[36] Merlo G Cozzani E Burlando M Calvieri S Potenza C Stingeni L. Effects of Tnfalpha Inhibitors in Patients With Psoriasis and Metabolic Syndrome: A Preliminary Study. G Ital Dermatol Venereol (2020) 155:14–8. 10.23736/S0392-0488.17.05621-8 28421729

[37] Botelho KP Pontes MAA Rodrigues CEM Freitas MVC. Prevalence of Metabolic Syndrome Among Patients With Psoriasis Treated With TNF Inhibitors and the Effects of Anti-TNF Therapy on Their Lipid Profile: A Prospective Cohort Study. Metab Syndr Relat Disord (2020) 18:154–60. 10.1089/met.2019.0092 31928509

[38] Arjuman A Chandra NC. Differential Pro-Inflammatory Responses of TNF-Alpha Receptors (TNFR1 and TNFR2) on LOX-1 Signalling. Mol Biol Rep (2015) 42:1039–47. 10.1007/s11033-014-3841-y 25416967

[39] Pina T Armesto S Lopez-Mejias R Genre F Ubilla B Gonzalez-Lopez MA. Anti-TNF-Alpha; Therapy Improves Insulin Sensitivity in Non-Diabetic Patients With Psoriasis: A 6-Month Prospective Study. J Eur Acad Dermatol Venereol: JEADV (2015) 29:1325–30. 10.1111/jdv.12814 25353352

[40] Antohe JL Bili A Sartorius JA Kirchner HL Morris SJ Dancea S. Diabetes Mellitus Risk in Rheumatoid Arthritis: Reduced Incidence With Anti-Tumor Necrosis Factor Alpha Therapy. Arthritis Care Res (Hoboken) (2012) 64:215–21. 10.1002/acr.20657 21972198

[41] Solomon DH Massarotti E Garg R Liu J Canning C Schneeweiss S. Association Between Disease-Modifying Antirheumatic Drugs and Diabetes Risk in Patients With Rheumatoid Arthritis and Psoriasis. JAMA (2011) 305:2525–31. 10.1001/jama.2011.878 21693740

[42] Broussard SR McCusker RH Novakofski JE Strle K Shen WH Johnson RW. IL-1beta Impairs Insulin-Like Growth Factor I-Induced Differentiation and Downstream Activation Signals of the Insulin-Like Growth Factor I Receptor in Myoblasts. J Immunol (2004) 172:7713–20. 10.4049/jimmunol.172.12.7713 15187154

[43] Rui L Aguirre V Kim JK Shulman GI Lee A Corbould A. Insulin/IGF-1 and TNF-Alpha Stimulate Phosphorylation of IRS-1 at Inhibitory Ser307 via Distinct Pathways. J Clin Invest (2001) 107:181–9. 10.1172/JCI10934 PMC199174 11160134

[44] Gisondi P Fostini AC Fossà I Girolomoni G Targher G. Psoriasis and the Metabolic Syndrome. Clinics Dermatol (2018) 36:21–8. 10.1016/j.clindermatol.2017.09.005 29241748

[45] Kofoed K Clemmensen A Mikkelsen UR Simonsen L Andersen O Gniadecki R. Effects of Anti-Tumor Necrosis Factor Therapy on Body Composition and Insulin Sensitivity in Patients With Psoriasis. Arch Dermatol (2012) 148:1089–91. 10.1001/archdermatol.2012.1753 22986877

[46] Fatima N Faisal SM Zubair S Siddiqui SS Moin S Owais M. Emerging Role of Interleukins IL-23/IL-17 Axis and Biochemical Markers in the Pathogenesis of Type 2 Diabetes: Association With Age and Gender in Human Subjects. Int J Biol Macromol (2017) 105:1279–88. 10.1016/j.ijbiomac.2017.07.155 28757426

[47] Goyal R Faizy AF Siddiqui SS Singhai M. Evaluation of TNF-Alpha and IL-6 Levels in Obese and Non-Obese Diabetics: Pre- and Postinsulin Effects. N Am J Med Sci (2012) 4:180–4. 10.4103/1947-2714.94944 PMC3334258 22536561

[48] Schmidt FM Weschenfelder J Sander C Minkwitz J Thormann J Chittka T. Inflammatory Cytokines in General and Central Obesity and Modulating Effects of Physical Activity. PloS One (2015) 10:e0121971. 10.1371/journal.pone.0121971 25781614 PMC4363366

[49] Wensveen FM Jelencic V Valentic S Sestan M Wensveen TT Theurich S. NK Cells Link Obesity-Induced Adipose Stress to Inflammation and Insulin Resistance. Nat Immunol (2015) 16:376–85. 10.1038/ni.3120 25729921

[50] Scheja L Heeren J. The Endocrine Function of Adipose Tissues in Health and Cardiometabolic Disease. Nat Rev Endocrinol (2019) 15:507–24. 10.1038/s41574-019-0230-6 31296970

[51] Yamauchi T Kamon J Ito Y Tsuchida A Yokomizo T Kita S. Cloning of Adiponectin Receptors That Mediate Antidiabetic Metabolic Effects. Nature (2003) 423:762–9. 10.1038/nature01705 12802337

[52] Li X Zhang D Vatner DF Goedeke L Hirabara SM Zhang Y. Mechanisms by Which Adiponectin Reverses High Fat Diet-Induced Insulin Resistance in Mice. Proc Natl Acad Sci USA (2020) 117:32584–93. 10.1073/pnas.1922169117 PMC7768680 33293421

[53] Yanai H Yoshida H. Beneficial Effects of Adiponectin on Glucose and Lipid Metabolism and Atherosclerotic Progression: Mechanisms and Perspectives. Int J Mol Sci (2019) 20:1–25. 10.3390/ijms20051190 PMC6429491 30857216

[54] Ahmad A Ali T Kim MW Khan A Jo MH Rehman SU. Adiponectin Homolog Novel Osmotin Protects Obesity/Diabetes-Induced NAFLD by Upregulating AdipoRs/PPARalpha Signaling in Ob/Ob and Db/Db Transgenic Mouse Models. Metabolism (2019) 90:31–43. 10.1016/j.metabol.2018.10.004 30473057

[55] Sereflican B Goksugur N Bugdayci G Polat M Parlak HA. Serum Visfatin, Adiponectin, and Tumor Necrosis Factor Alpha (TNF-Alpha) Levels in Patients With Psoriasis and Their Correlation With Disease Severity. Acta Dermatovenerol Croat (2016) 24:13–9. 27149125

[56] He Y Lu L Wei X Jin D Qian T Yu A. The Multimerization and Secretion of Adiponectin Are Regulated by TNF-Alpha. Endocrine (2016) 51:456–68. 10.1007/s12020-015-0741-4 26407855

[57] Morris EV Suchacki KJ Hocking J Cartwright R Sowman A Gamez B. Myeloma Cells Down-Regulate Adiponectin in Bone Marrow Adipocytes via TNF-Alpha. J Bone Miner Res (2020) 35:942–55. 10.1002/jbmr.3951 PMC9328417 31886918

[58] Kong Y Zhang S Wu R Su X Peng D Zhao M. New Insights Into Different Adipokines in Linking the Pathophysiology of Obesity and Psoriasis. Lipids Health Dis (2019) 18:171. 10.1186/s12944-019-1115-3 31521168 PMC6745073

[59] Bai F Zheng W Dong Y Wang J Garstka MA Li R. Serum Levels of Adipokines and Cytokines in Psoriasis Patients: A Systematic Review and Meta-Analysis. Oncotarget (2018) 9:1266–78. 10.18632/oncotarget.22260 PMC5787437 29416693

[60] Langley RG Ellis CN. Evaluating Psoriasis With Psoriasis Area and Severity Index, Psoriasis Global Assessment, and Lattice System Physician’s Global Assessment. J Am Acad Dermatol (2004) 51:563–9. 10.1016/j.jaad.2004.04.012 15389191

[61] Gerdes S Pinter A Biermann M Papavassilis C Reinhardt M. Adiponectin Levels in a Large Pooled Plaque Psoriasis Study Population. J Dermatolog Treat (2020) 31:531–4. 10.1080/09546634.2019.1621979 31179792

[62] Chan WSA Liew CF Theng CTS Oon HH. Serum Adiponectin Levels and Their Association With Cardiometabolic Risk Factors in Patients With Psoriasis. Cureus (2020) 12:e8128. 10.7759/cureus.8128 32550048 PMC7294861

[63] Landecho MF Tuero C Valenti V Bilbao I de la Higuera M Fruhbeck G. Relevance of Leptin and Other Adipokines in Obesity-Associated Cardiovascular Risk. Nutrients (2019) 11:1–16. 10.3390/nu11112664 PMC6893824 31694146

[64] Ghadge AA Khaire AA. Leptin as a Predictive Marker for Metabolic Syndrome. Cytokine (2019) 121:154735. 10.1016/j.cyto.2019.154735 31154250

[65] Zhao S Zhu Y Schultz RD Li N He Z Zhang Z. Partial Leptin Reduction as an Insulin Sensitization and Weight Loss Strategy. Cell Metab (2019) 30:706–19.e706. 10.1016/j.cmet.2019.08.005 31495688 PMC6774814

[66] Kyriakou A Patsatsi A Sotiriadis D Goulis DG. Serum Leptin, Resistin, and Adiponectin Concentrations in Psoriasis: A Meta-Analysis of Observational Studies. Dermatology (2017) 233:378–89. 10.1159/000481882 29232663

[67] Coimbra S Oliveira H Reis F Belo L Rocha S Quintanilha A. Circulating Adipokine Levels in Portuguese Patients With Psoriasis Vulgaris According to Body Mass Index, Severity and Therapy. J Eur Acad Dermatol Venereol: JEADV (2010) 24:1386–94. 10.1111/j.1468-3083.2010.03647.x 20337818

[68] Karadag AS Ertugrul DT Kalkan G Bilgili SG Celik HT Takci Z. The Effect of Acitretin Treatment on Insulin Resistance, Retinol-Binding Protein-4, Leptin, and Adiponectin in Psoriasis Vulgaris: A Noncontrolled Study. Dermatology (2013) 227:103–8. 10.1159/000351769 24021889

[69] Hwang J Yoo JA Yoon H Han T Yoon J An S. The Role of Leptin in the Association Between Obesity and Psoriasis. Biomol Ther (Seoul) (2021) 29:11–21. 10.4062/biomolther.2020.054 32690821 PMC7771847

[70] Bavoso NC Pinto JM Soares MMS Diniz MDS Junior TAL. Psoriasis in Obesity: Comparison of Serum Levels of Leptin and Adiponectin in Obese Subjects - Cases and Controls. Bras Dermatol (2019) 94:192–7. 10.1590/abd1806-4841.20197716 PMC6486065 31090824

[71] Liang L Hur J Kang JY Rhee CK Kim YK Lee SY. Effect of the Anti-IL-17 Antibody on Allergic Inflammation in an Obesity-Related Asthma Model. Korean J Intern Med (2018) 33:1210–23. 10.3904/kjim.2017.207 PMC6234391 29665658

[72] Finkel T. Signal Transduction by Reactive Oxygen Species. J Cell Biol (2011) 194:7–15. 10.1083/jcb.201102095 21746850 PMC3135394

[73] Darroudi S Fereydouni N Tayefi M Ahmadnezhad M Zamani P Tayefi B. Oxidative Stress and Inflammation, Two Features Associated With a High Percentage Body Fat, and That May Lead to Diabetes Mellitus and Metabolic Syndrome. Biofactors (2019) 45:35–42. 10.1002/biof.1459 30561055

[74] Furukawa S Fujita T Shimabukuro M Iwaki M Yamada Y Nakajima Y. Increased Oxidative Stress in Obesity and Its Impact on Metabolic Syndrome. J Clin Invest (2004) 114:1752–61. 10.1172/JCI21625 PMC535065 15599400

[75] Houstis N Rosen ED Lander ES. Reactive Oxygen Species Have a Causal Role in Multiple Forms of Insulin Resistance. Nature (2006) 440:944–8. 10.1038/nature04634 16612386

[76] Onyango AN. Cellular Stresses and Stress Responses in the Pathogenesis of Insulin Resistance. Oxid Med Cell Longev (2018) 2018:4321714. 10.1155/2018/4321714 30116482 PMC6079365

[77] Hofmanis J Hofmane D Svirskis S Mackevics V Tretjakovs P Lejnieks A. HDL-C Role in Acquired Aortic Valve Stenosis Patients and Its Relationship With Oxidative Stress. Medicina (Kaunas) (2019) 55:1–16. 10.3390/medicina55080416 PMC6723197 31362438

[78] Bastard JP Couffignal C Fellahi S Bard JM Mentre F Salmon D. Diabetes and Dyslipidaemia Are Associated With Oxidative Stress Independently of Inflammation in Long-Term Antiretroviral-Treated HIV-Infected Patients. Diabetes Metab (2019) 45:573–81. 10.1016/j.diabet.2019.02.008 30862472

[79] Gao M Zhao Z Lv P Li Y Gao J Zhang M. Quantitative Combination of Natural Anti-Oxidants Prevents Metabolic Syndrome by Reducing Oxidative Stress. Redox Biol (2015) 6:206–17. 10.1016/j.redox.2015.06.013 PMC4536297 26262997

[80] Vona R Gambardella L Cittadini C Straface E Pietraforte D. Biomarkers of Oxidative Stress in Metabolic Syndrome and Associated Diseases. Oxid Med Cell Longev (2019) 2019:8267234. 10.1155/2019/8267234 31191805 PMC6525823

[81] Ferretti G Bacchetti T Campanati A Simonetti O Liberati G Offidani A. Correlation Between Lipoprotein(a) and Lipid Peroxidation in Psoriasis: Role of the Enzyme Paraoxonase-1. Br J Dermatol (2012) 166:204–7. 10.1111/j.1365-2133.2011.10539.x 21790517

[82] Nemati H Khodarahmi R Sadeghi M Ebrahimi A Rezaei M Vaisi-Raygani A. Antioxidant Status in Patients With Psoriasis. Cell Biochem Funct (2014) 32:268–73. 10.1002/cbf.3011 24895696

[83] Sikar Akturk A Ozdogan HK Bayramgurler D Cekmen MB Bilen N Kiran R. Nitric Oxide and Malondialdehyde Levels in Plasma and Tissue of Psoriasis Patients. J Eur Acad Dermatol Venereol: JEADV (2012) 26:833–7. 10.1111/j.1468-3083.2011.04164.x 21749467

[84] Ambrozewicz E Wojcik P Wronski A Luczaj W Jastrzab A Zarkovic N. Pathophysiological Alterations of Redox Signaling and Endocannabinoid System in Granulocytes and Plasma of Psoriatic Patients. Cells (2018) 7:1–18. 10.3390/cells7100159 PMC6210326 30301214

[85] Wojcik P Gegotek A Wronski A Jastrzab A Zebrowska A Skrzydlewska E. Effect of Redox Imbalance on Protein Modifications in Lymphocytes of Psoriatic Patients. J Biochem (2020) 167:323–31. 10.1093/jb/mvz096 31710683

[86] Plenkowska J Gabig-Ciminska M Mozolewski P. Oxidative Stress as an Important Contributor to the Pathogenesis of Psoriasis. Int J Mol Sci (2020) 21:1–15. 10.3390/ijms21176206 PMC7503883 32867343

[87] Oszukowska M Kozlowska M Kaszuba A. Paraoxonase-1 and Other Factors Related to Oxidative Stress in Psoriasis. Postepy Dermatol Alergol (2020) 37:92–6. 10.5114/ada.2020.93386 PMC7247073 32467691

[88] Lin X Huang T. Oxidative Stress in Psoriasis and Potential Therapeutic Use of Antioxidants. Free Radic Res (2016) 50:585–95. 10.3109/10715762.2016.1162301 27098416

[89] Liu A Zhao W Zhang B Tu Y Wang Q Li J. Cimifugin Ameliorates Imiquimod-Induced Psoriasis by Inhibiting Oxidative Stress and Inflammation *via* NF-KappaB/MAPK Pathway. Biosci Rep (2020) 40:1–11. 10.1042/BSR20200471 PMC7300284 32515468

[90] Yazici C Kose K Utas S Tanrikulu E Taslidere N. A Novel Approach in Psoriasis: First Usage of Known Protein Oxidation Markers to Prove Oxidative Stress. Arch Dermatol Res (2016) 308:207–12. 10.1007/s00403-016-1624-0 26842230

[91] Rowland AA Voeltz GK. Endoplasmic Reticulum-Mitochondria Contacts: Function of the Junction. Nat Rev Mol Cell Biol (2012) 13:607–25. 10.1038/nrm3440 PMC5111635 22992592

[92] Fewell SW Travers KJ Weissman JS Brodsky JL. The Action of Molecular Chaperones in the Early Secretory Pathway. Annu Rev Genet (2001) 35:149–91. 10.1146/annurev.genet.35.102401.090313 11700281

[93] S. Mohan PRMR Brown L Ayyappan P K. G R. Endoplasmic Reticulum Stress: A Master Regulator of Metabolic Syndrome. Eur J Pharmacol (2019) 860:172553. 10.1016/j.ejphar.2019.172553 31325433

[94] Reid DW Nicchitta CV. Diversity and Selectivity in Mrna Translation on the Endoplasmic Reticulum. Nat Rev Mol Cell Biol (2015) 16:221–31. 10.1038/nrm3958 PMC4494666 25735911

[95] Schwarz DS Blower MD. The Endoplasmic Reticulum: Structure, Function and Response to Cellular Signaling. Cell Mol Life Sci (2016) 73:79–94. 10.1007/s00018-015-2052-6 26433683 PMC4700099

[96] Fagone P Jackowski S. Membrane Phospholipid Synthesis and Endoplasmic Reticulum Function. J Lipid Res (2009) 50 Suppl:S311–6. 10.1194/jlr.R800049-JLR200 PMC2674712 18952570

[97] Hetz C. The Unfolded Protein Response: Controlling Cell Fate Decisions Under ER Stress and Beyond. Nat Rev Mol Cell Biol (2012) 13:89–102. 10.1038/nrm3270 22251901

[98] Ozcan U Cao Q Yilmaz E Lee AH Iwakoshi NN Ozdelen E. Endoplasmic Reticulum Stress Links Obesity, Insulin Action, and Type 2 Diabetes. Science (2004) 306:457–61. 10.1126/science.1103160 15486293

[99] Wang M Kaufman RJ. Protein Misfolding in the Endoplasmic Reticulum as a Conduit to Human Disease. Nature (2016) 529:326–35. 10.1038/nature17041 26791723

[100] Shan B Wang X Wu Y Xu C Xia Z Dai J. The Metabolic ER Stress Sensor IRE1alpha Suppresses Alternative Activation of Macrophages and Impairs Energy Expenditure in Obesity. Nat Immunol (2017) 18:519–29. 10.1038/ni.3709 28346409

[101] Radwan E Bakr MH Taha S Sayed SA Farrag AA Ali M. Inhibition of Endoplasmic Reticulum Stress Ameliorates Cardiovascular Injury in a Rat Model of Metabolic Syndrome. J Mol Cell Cardiol (2020) 143:15–25. 10.1016/j.yjmcc.2020.04.020 32311415

[102] Liu F Zhu S Ni L Huang L Wang K Zhou Y. Dexmedetomidine Alleviates Insulin Resistance in Hepatocytes by Reducing Endoplasmic Reticulum Stress. Endocrine (2020) 67:87–94. 10.1007/s12020-019-02118-1 31679138 PMC6969002

[103] Jia B Wang Y Yu G Cheng Y Yang C Cao F. Naringenin Ameliorates Insulin Resistance by Modulating Endoplasmic Reticulum Stress in Hepatitis C Virus-Infected Liver. BioMed Pharmacother (2019) 115:108848. 10.1016/j.biopha.2019.108848 31039496

[104] Zhao H Zhang Y Shu L Song G Ma H. Resveratrol Reduces Liver Endoplasmic Reticulum Stress and Improves Insulin Sensitivity *in Vivo* and *in Vitro* . Drug Des Devel Ther (2019) 13:1473–85. 10.2147/DDDT.S203833 PMC6505469 31118581

[105] Liang B Liu L Huang H Li L Zhou J. High T3 Induces Beta-Cell Insulin Resistance *via* Endoplasmic Reticulum Stress. Mediators Inflammation (2020) 2020:5287108. 10.1155/2020/5287108 PMC7396010 32774144

[106] Brown M Dainty S Strudwick N Mihai AD Watson JN Dendooven R. Endoplasmic Reticulum Stress Causes Insulin Resistance by Inhibiting Delivery of Newly Synthesized Insulin Receptors to the Cell Surface. Mol Biol Cell (2020) 31:2597–629. 10.1091/mbc.E18-01-0013 PMC7851869 32877278

[107] Xue X Piao JH Nakajima A Sakon-Komazawa S Kojima Y Mori K. Tumor Necrosis Factor Alpha (TNFalpha) Induces the Unfolded Protein Response (UPR) in a Reactive Oxygen Species (ROS)-Dependent Fashion, and the UPR Counteracts ROS Accumulation by TNFalpha. J Biol Chem (2005) 280:33917–25. 10.1074/jbc.M505818200 16107336

[108] Hasnain SZ Lourie R Das I Chen AC McGuckin MA. The Interplay Between Endoplasmic Reticulum Stress and Inflammation. Immunol Cell Biol (2012) 90:260–70. 10.1038/icb.2011.112 PMC7165805 22249202

[109] Kim SR Kim HJ Kim DI Lee KB Park HJ Jeong JS. Blockade of Interplay Between IL-17A and Endoplasmic Reticulum Stress Attenuates LPS-Induced Lung Injury. Theranostics (2015) 5:1343–62. 10.7150/thno.11685 PMC4615737 26516372

[110] Yang Z Liu Q Shi H Jiang X Wang S Lu Y. Interleukin 17A Exacerbates ER-Stress-Mediated Inflammation of Macrophages Following ICH. Mol Immunol (2018) 101:38–45. 10.1016/j.molimm.2018.05.020 29859495

[111] Zhao M Luo J Xiao B Tang H Song F Ding X. Endoplasmic Reticulum Stress Links Psoriasis Vulgaris With Keratinocyte Inflammation. Postepy Dermatol Alergol (2020) 37:34–40. 10.5114/ada.2020.93382 32467681 PMC7247056

[112] Shimoura N Nagai H Fujiwara S Jimbo H Nishigori C. Exacerbation and Prolongation of Psoriasiform Inflammation in Diabetic Obese Mice: A Synergistic Role of CXCL5 and Endoplasmic Reticulum Stress. J Invest Dermatol (2018) 138:854–63. 10.1016/j.jid.2017.10.023 29111234

[113] Ruff WE Kriegel MA. Autoimmune Host-Microbiota Interactions at Barrier Sites and Beyond. Trends Mol Med (2015) 21:233–44. 10.1016/j.molmed.2015.02.006 PMC5918312 25771098

[114] Cho I Blaser MJ. The Human Microbiome: At the Interface of Health and Disease. Nat Rev Genet (2012) 13:260–70. 10.1038/nrg3182 PMC3418802 22411464

[115] Lynch SV Pedersen O. The Human Intestinal Microbiome in Health and Disease. N Engl J Med (2016) 375:2369–79. 10.1056/NEJMra1600266 27974040

[116] Wu H Esteve E Tremaroli V Khan MT Caesar R Manneras-Holm L. Metformin Alters the Gut Microbiome of Individuals With Treatment-Naive Type 2 Diabetes, Contributing to the Therapeutic Effects of the Drug. Nat Med (2017) 23:850–8. 10.1038/nm.4345 28530702

[117] Virtue AT McCright SJ Wright JM Jimenez MT Mowel WK Kotzin JJ. The Gut Microbiota Regulates White Adipose Tissue Inflammation and Obesity via a Family of MicroRNAs. Sci Transl Med (2019) 11:1–15. 10.1126/scitranslmed.aav1892 PMC7050429 31189717

[118] Macchione IG Lopetuso LR Ianiro G Napoli M Gibiino G Rizzatti G. Akkermansia Muciniphila: Key Player in Metabolic and Gastrointestinal Disorders. Eur Rev Med Pharmacol Sci (2019) 23:8075–83. 10.26355/eurrev_201909_19024 31599433

[119] Zhang T Li Q Cheng L Buch H Zhang F. Akkermansia Muciniphila Is a Promising Probiotic. Microb Biotechnol (2019) 12:1109–25. 10.1111/1751-7915.13410 PMC6801136 31006995

[120] Cani PD de Vos WM. Next-Generation Beneficial Microbes: The Case of Akkermansia Muciniphila. Front Microbiol (2017) 8:1765. 10.3389/fmicb.2017.01765 29018410 PMC5614963

[121] Depommier C Everard A Druart C Plovier H Van Hul M Vieira-Silva S. Supplementation With Akkermansia Muciniphila in Overweight and Obese Human Volunteers: A Proof-of-Concept Exploratory Study. Nat Med (2019) 25:1096–103. 10.1038/s41591-019-0495-2 PMC6699990 31263284

[122] Codoner FM Ramirez-Bosca A Climent E Carrion-Gutierrez M Guerrero M Perez-Orquin JM. Gut Microbial Composition in Patients With Psoriasis. Sci Rep (2018) 8:3812. 10.1038/s41598-018-22125-y 29491401 PMC5830498

[123] Chen YJ Ho HJ Tseng CH Lai ZL Shieh JJ Wu CY. Intestinal Microbiota Profiling and Predicted Metabolic Dysregulation in Psoriasis Patients. Exp Dermatol (2018) 27:1336–43. 10.1111/exd.13786 30238519

[124] Shapiro J Cohen NA Shalev V Uzan A Koren O Maharshak N. Psoriatic Patients Have a Distinct Structural and Functional Fecal Microbiota Compared With Controls. J Dermatol (2019) 46:595–603. 10.1111/1346-8138.14933 31141234

[125] Tan L Zhao S Zhu W Wu L Li J Shen M. The Akkermansia Muciniphila Is a Gut Microbiota Signature in Psoriasis. Exp Dermatol (2018) 27:144–9. 10.1111/exd.13463 29130553

[126] Chen L Li J Zhu W Kuang Y Liu T Zhang W. Skin and Gut Microbiome in Psoriasis: Gaining Insight Into the Pathophysiology of It and Finding Novel Therapeutic Strategies. Front Microbiol (2020) 11:589726. 10.3389/fmicb.2020.589726 33384669 PMC7769758

[127] Chelakkot C Ghim J Ryu SH. Mechanisms Regulating Intestinal Barrier Integrity and Its Pathological Implications. Exp Mol Med (2018) 50:103. 10.1038/s12276-018-0126-x PMC6095905 30115904

[128] Cox AJ Zhang P Bowden DW Devereaux B Davoren PM Cripps AW. Increased Intestinal Permeability as a Risk Factor for Type 2 Diabetes. Diabetes Metab (2017) 43:163–6. 10.1016/j.diabet.2016.09.004 27745826

[129] Sorini C Cosorich I Lo Conte M De Giorgi L Facciotti F Luciano R. Loss of Gut Barrier Integrity Triggers Activation of Islet-Reactive T Cells and Autoimmune Diabetes. Proc Natl Acad Sci USA (2019) 116:15140–9. 10.1073/pnas.1814558116 PMC6660755 31182588

[130] Damms-Machado A Louis S Schnitzer A Volynets V Rings A Basrai M. Gut Permeability Is Related to Body Weight, Fatty Liver Disease, and Insulin Resistance in Obese Individuals Undergoing Weight Reduction. Am J Clin Nutr (2017) 105:127–35. 10.3945/ajcn.116.131110 28049662

[131] Ma L Ni Y Wang Z Tu W Ni L Zhuge F. Spermidine Improves Gut Barrier Integrity and Gut Microbiota Function in Diet-Induced Obese Mice. Gut Microbes (2020) 12:1–19. 10.1080/19490976.2020.1832857 PMC7668533 33151120

[132] Mirza A Mao-Draayer Y. The Gut Microbiome and Microbial Translocation in Multiple Sclerosis. Clin Immunol (2017) 183:213–24. 10.1016/j.clim.2017.03.001 28286112

[133] Boulange CL Neves AL Chilloux J Nicholson JK Dumas ME. Impact of the Gut Microbiota on Inflammation, Obesity, and Metabolic Disease. Genome Med (2016) 8:42. 10.1186/s13073-016-0303-2 27098727 PMC4839080

[134] Okubo Y Oki N Takeda H Amaya M Ito S Osada M. Increased Microorganisms DNA Levels in Peripheral Blood Monocytes From Psoriatic Patients Using PCR With Universal Ribosomal RNA Primers. J Dermatol (2002) 29:547–55. 10.1111/j.1346-8138.2002.tb00179.x 12392062

[135] Munz OH Sela S Baker BS Griffiths CE Powles AV Fry L. Evidence for the Presence of Bacteria in the Blood of Psoriasis Patients. Arch Dermatol Res (2010) 302:495–8. 10.1007/s00403-010-1065-0 20607546

[136] Sikora M Chrabaszcz M Maciejewski C Zaremba M Waskiel A Olszewska M. Intestinal Barrier Integrity in Patients With Plaque Psoriasis. J Dermatol (2018) 45:1468–70. 10.1111/1346-8138.14647 30222202

[137] Sikora M Stec A Chrabaszcz M Waskiel-Burnat A Zaremba M Olszewska M. Intestinal Fatty Acid Binding Protein, a Biomarker of Intestinal Barrier, Is Associated With Severity of Psoriasis. J Clin Med (2019) 8:1–9. 10.3390/jcm8071021 PMC6678629 31336842

[138] Visser MJE Kell DB Pretorius E. Bacterial Dysbiosis and Translocation in Psoriasis Vulgaris. Front Cell Infect Microbiol (2019) 9:7. 10.3389/fcimb.2019.00007 30778377 PMC6369634

[139] Welty FK Alfaddagh A Elajami TK. Targeting Inflammation in Metabolic Syndrome. Trans research: J Lab Clin Med (2016) 167:257–80. 10.1016/j.trsl.2015.06.017 PMC6800061 26207884

[140] Ghemrawi R Battaglia-Hsu SF Arnold C. Endoplasmic Reticulum Stress in Metabolic Disorders. Cells (2018) 7:1–39. 10.3390/cells7060063 PMC6025008 29921793

[141] Rani V Deep G Singh RK Palle K Yadav UC. Oxidative Stress and Metabolic Disorders: Pathogenesis and Therapeutic Strategies. Life Sci (2016) 148:183–93. 10.1016/j.lfs.2016.02.002 26851532

